# Primary Pulmonary Epithelioid Angiosarcoma Presenting in a 59-Year-Old Woman: A Report of a Rare Case

**DOI:** 10.7759/cureus.69191

**Published:** 2024-09-11

**Authors:** Muhammad Tahir, Ardenne Martin, Carlina Madelaire, Guillermo A Herrera, Carl Maltese, Rodney E Shackelford

**Affiliations:** 1 Pathology and Laboratory Medicine, University of South Alabama College of Medicine, Mobile, USA; 2 Pathology, University of South Alabama College of Medicine, Mobile, USA; 3 Cardiothoracic Surgery, University of South Alabama College of Medicine, Mobile, USA

**Keywords:** angiosarcoma, epithelioid angiosarcoma, lung sarcoma, primary angiosarcoma, pulmonary epithelioid angiosarcoma

## Abstract

Primary pulmonary angiosarcomas are rare malignancies, with aggressive clinical behavior and poor prognosis. Here we present a case of a rare primary pulmonary epithelioid angiosarcoma in a 59-year-old woman who initially presented with right-sided chest pain and shortness of breath. Chest X-ray revealed right lower lobe atelectasis, while a chest computed tomography angiography (CTA) showed a large right hydrothorax with collapse of most of the right lung. A right lower lobe resection was performed and histologic and immunohistochemical analyses were consistent with a primary pulmonary epithelioid angiosarcoma. The patient was discharged, given supportive care, and died 12 days following her last operation.

## Introduction

Angiosarcomas are rare, highly aggressive sarcomas arising from lymphatic or endothelial cells [1]. This vascular tumor is so rare that only 30 cases have been reported in the English literature since the first described case in 1923. These tumors account for less than 2% of sarcomas, are most common in adults and the elderly, and can occur in any location within the body, most frequently the superficial tissues of the head and neck and less frequently, in the soft tissues, viscera, and retroperitoneum [1]. Pulmonary angiosarcomas are predominantly (>90%) metastases from primary malignancies of the skin, liver, bone, breast, or more rarely, the heart [1-3]. Primary pulmonary angiosarcomas are rare malignancies, with only about 30 reported cases [2-4]. Here we describe a rare case of a primary pulmonary angiosarcoma in a 59-year-old woman. We present this case to highlight the diagnostic challenges and clinical course of primary pulmonary epithelioid angiosarcoma, a rare and aggressive malignancy.

## Case presentation

**Figure 1:**
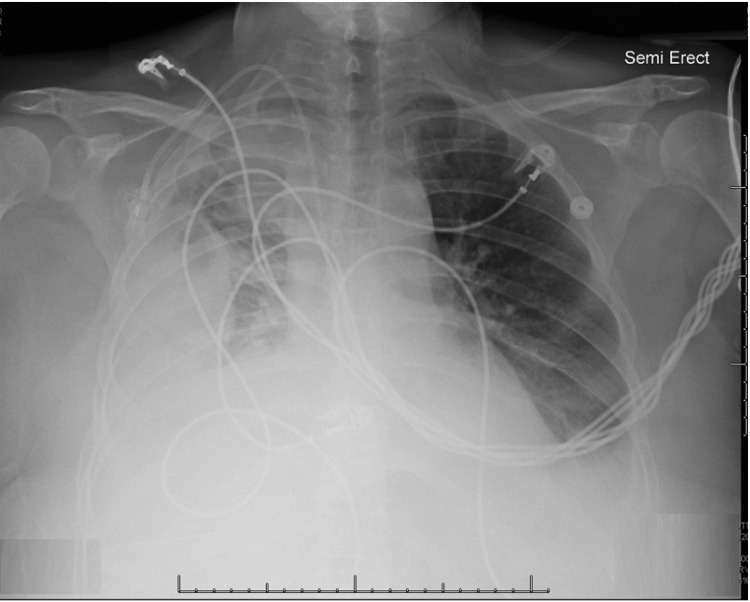
A chest X-ray showing a right lobe pleural effusion with near complete right-sided opacification, without a definitive pneumothorax. A much smaller pleural effusion is also seen on the left lung. Both lungs showed stable lucencies consistent with known pneumatoceles.

A 59-year-old Caucasian woman presented to the freestanding emergency department with a history of sharp, right-sided chest pain and increasing shortness of breath over a six-day period. Her past medical history included type II diabetes, hypertension, hyperlipidemia, rheumatologic disease (type unspecified; symptoms of joint pain and rash), non-alcoholic fatty liver disease, varicose veins, and prior COVID-19 infections in 2020 and 2021. A chest X-ray revealed right lobe pleural effusion with near complete opacification, without a definitive pneumothorax. A much smaller pleural effusion was also noted in the left lung. Both lungs showed stable luncencies consistent with known pneumatoceles (Figure 1).

**Figure 2:**
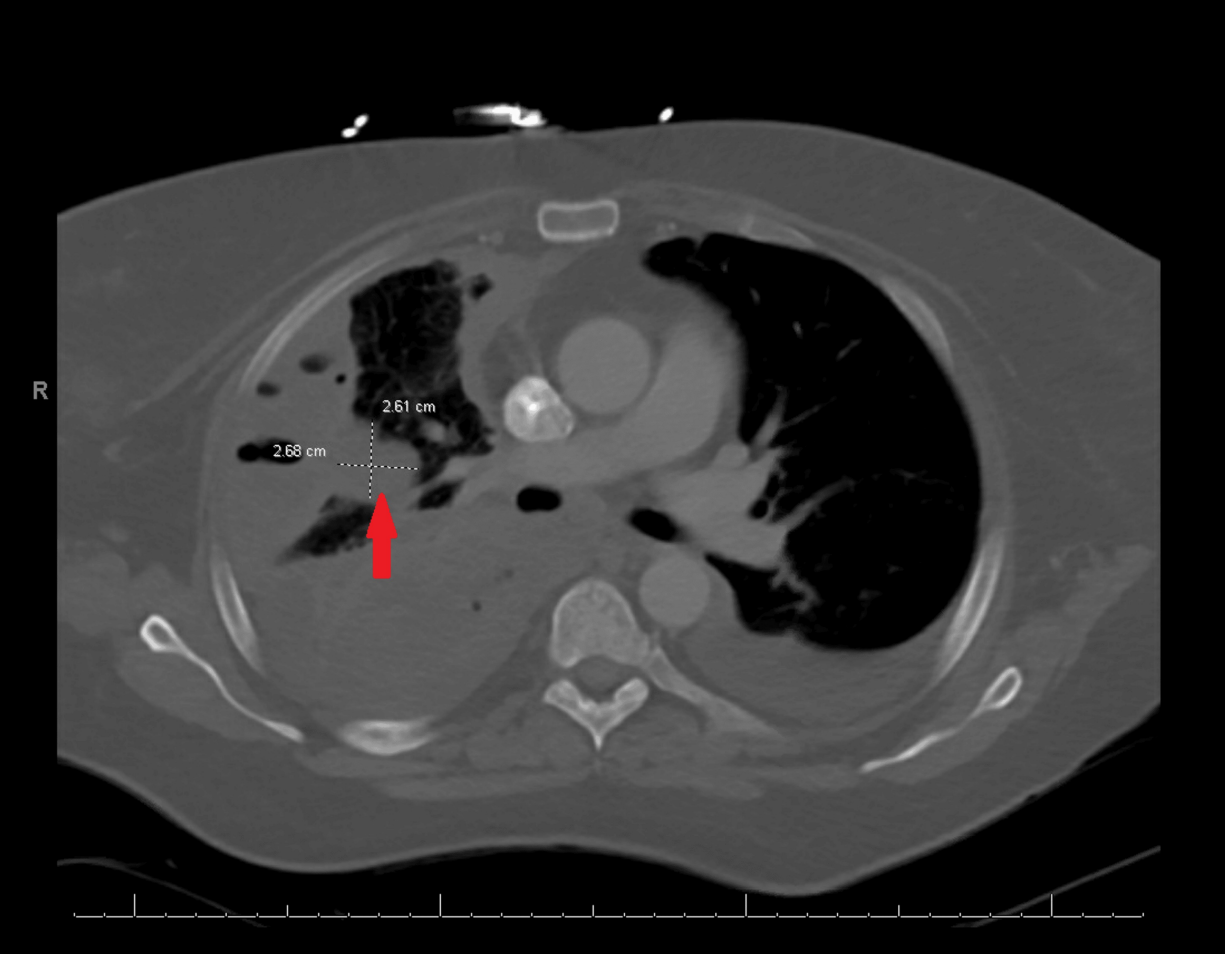
A computed tomography angiography of the patient’s chest showing a large right hydrothorax with a mediastinal shift to the left and a poorly defined mass-like right lung consolidation (arrow).

She was transferred to the University Hospital for further evaluation. Initial laboratory investigations revealed a significantly elevated D-dimer of 9.63 DDU (D-dimer units), so a chest computed tomography angiography (CTA) for a pulmonary embolism was performed. This revealed a large right hydrothorax with a collapse of most of the right lung, accompanied by a mediastinal shift to the left, without evidence of a pulmonary embolism. A poorly defined mass-like consolidation in the right lung was identified with an enlarging multivacuolated complex pleural effusion with scattered foci of air, right-sided chest wall edematous changes, and lytic erosions on 7th and 9th ribs. Lastly, small left pleural effusions were seen (Figure 2). Chest computed tomography revealed no masses or other unusual effusions outside of the lungs, including within the liver.

**Figure 3:**
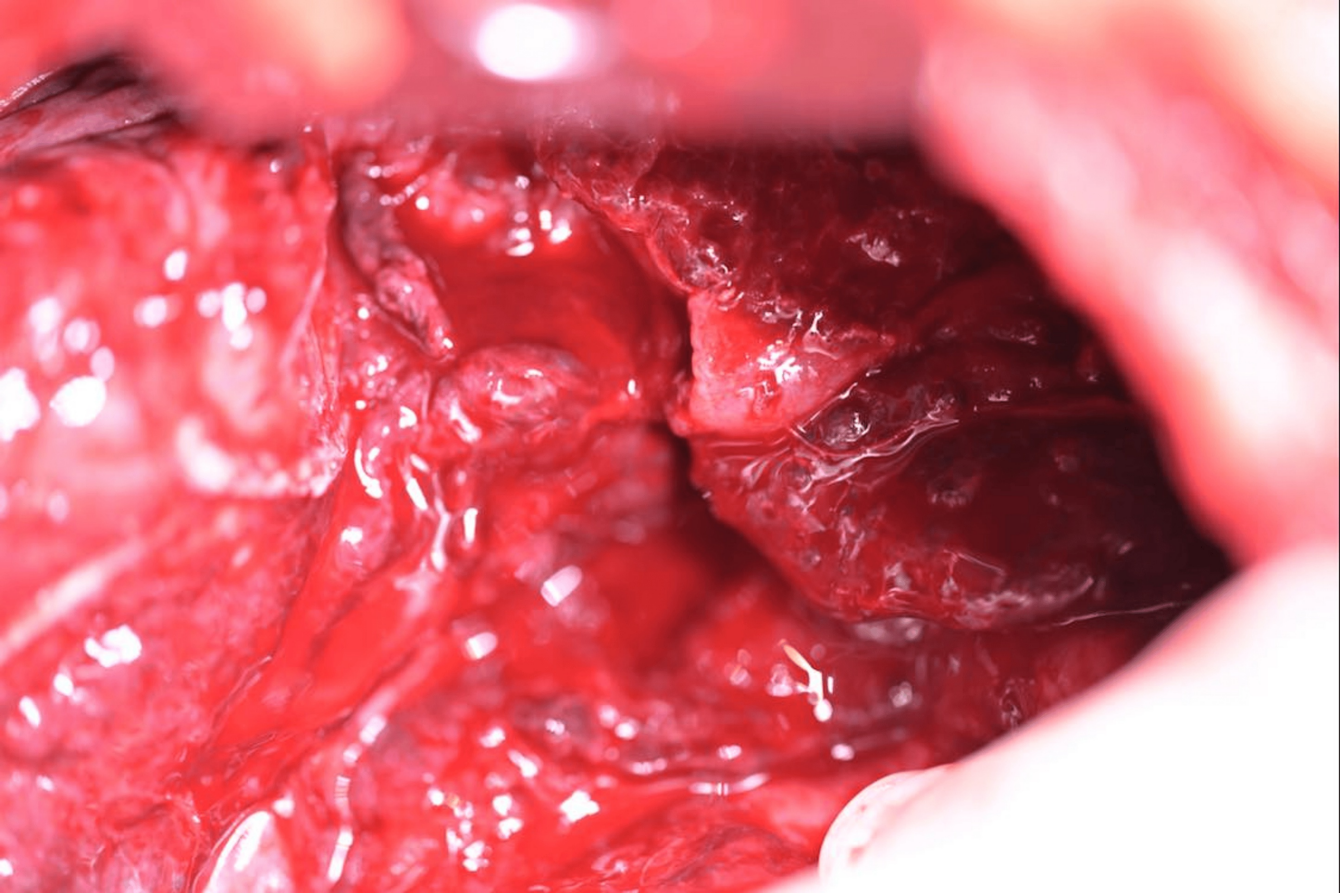
A photograph of the right lobectomy taken during surgery showing the thick hemorrhagic gelatinous oozing material that covered and filled much of the right lung and filled the right pleural cavity.

An initial complete blood count (CBC) revealed hemoglobin of 7.3 mg/dl (12-15 mg/dl) with hematocrit of 24.4%, (36-44%) repeat CBC showed hemoglobin of 6.3 mg/dl (12-15 mg/dl) hematocrit 21% (36-44%). There was concern for pneumonia, so she was treated with intravenous (IV) cefepime and vancomycin. The patient underwent a right lower lobe thoracotomy with wedge resection (Figure 3).

**Figure 4:**
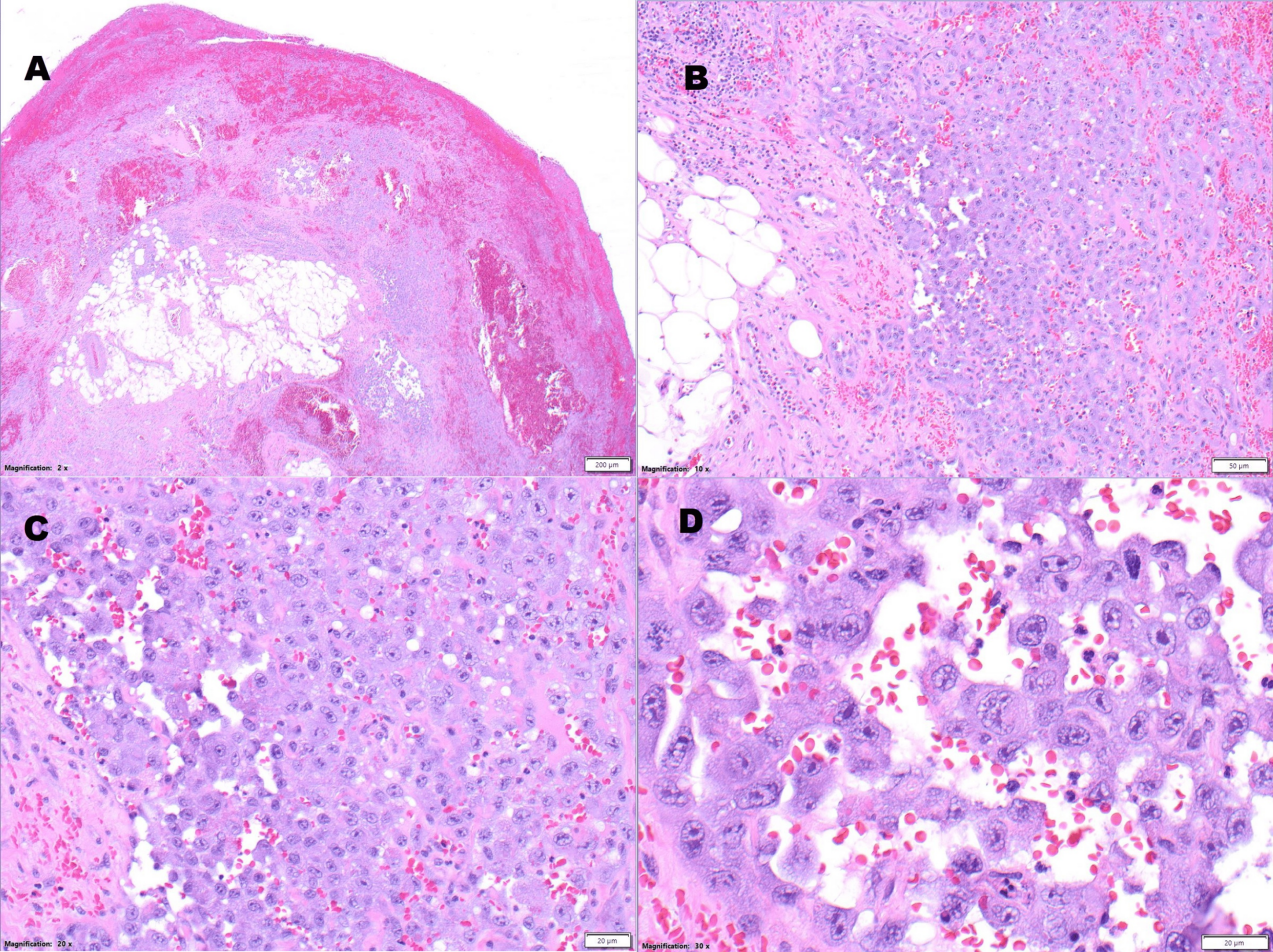
H&E sections of the right pulmonary lesion; magnifications (A) 2X, (B) 10X, (C) 20X, and (D) 30X. H&E: Hematoxylin and eosin.

A frozen section analysis was performed and a preliminary diagnosis of “highly suspicious for malignancy” was made. During the surgery, it was noted that the right lung parenchyma and pleural cavity were filled with thick hemorrhagic gelatinous oozing material. Histopathological examination of hematoxylin and eosin (H&E) sections of the resected pulmonary lobectomy revealed sheets of pleomorphic, polygonal to spindle-shaped cells forming layers and irregular blood-filled vascular channels which infiltrated into the pleura and throughout the right pulmonary parenchyma. A high mitotic index was seen, accompanied by focal necrosis (Figure 4).

**Figure 5:**
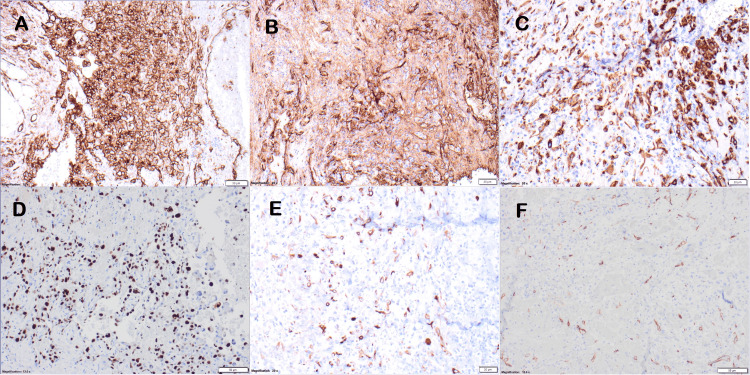
Immunohistochemical analysis showing some of the stains performed of the right pulmonary lesion; (A) CD31, (B) Factor VIII, (C) pan-cytokeratin (AE1/AE3), (D) Ki-67, (E) CK7, and (F) CD34, 20X.

The tumor was staged as pT1bpN (no nodes taken). Immunohistochemical analyses showed that the malignant cells were immunoreactive to CD31 and Factor VIII, focally reactive to AE1/AE3 and Ki-67. The tumor cells were immunonegative for D2-40, TTF-1, CK7, and CD34 (Figure 5). Based on histopathology and immunohistochemistry, a diagnosis of epithelioid angiosarcoma was made

Subsequently, two right thoracotomies were performed, and a right chest tube was placed over the next two weeks, while the patient experienced a persistent pneumothorax, severe pain at 7/10, and shortness of breath. The patient was sent to hospice care and died peacefully 12 days after her last operation.

## Discussion

Primary pulmonary angiosarcomas are rare malignancies, with the first example described in 1923 [2-5]. They most often arise in the pulmonary arteries, originating from lymphatic or vascular endothelial cells. Angiosarcomas can occur in any location in the body and are most frequently seen in the skin of the head and neck, the soft tissues, visceral organs, bone, and retroperitoneum [1-5]. Primary pulmonary angiosarcomas are rare with only about 30 cases recorded, resulting in an incidence of about 0.0001-0.030% in the general population [4,6]. They are most often seen in middle-aged men with an average age of 55.9 years at diagnosis and a survival time of about 12 months [4,6]. These tumors show aggressive growth characterized by extensive local invasion, local recurrence, and hematogenous metastasis. Most present with initial non-specific constitutional and respiratory symptoms, including cough, hemoptysis, shortness of breath, chest pain, pneumothorax, cyanosis, and weight loss, with the first two symptoms being most common [1-6]. As these symptoms are relatively non-specific, the initial diagnosis of a primary pulmonary angiosarcoma can be difficult. Often the initial differential diagnosis includes a thromboembolism, as was seen in the case present here, or other malignancies such as epithelial carcinomas and anaplastic melanomas [1,3]. Additionally, individuals presenting with primary pulmonary angiosarcoma typically lack the common risk factors for lung cancer, such as a history of tobacco use [1-4,6,7]. 

The diagnosis of primary pulmonary angiosarcoma requires the use of multiple modalities, including chest X-ray, CT, and MRI scans. The latter two have high sensitivity in discriminating angiosarcomas from other malignancies [1]. There also must be evidence that that the angiosarcoma is not a metastasis from another site, as was found in this case. The definitive diagnosis of a primary pulmonary angiosarcoma requires histologic and immunohistochemical analyses. By H&E staining, these tumors consist of polygonal to spindle-shaped to epithelioid primitive cells that form sheets and irregularly shaped anastomosing blood-filled vascular channels, exhibiting an often-high mitotic index, sometimes accompanied by focal necrosis and intratumoral hemorrhage. Poorly-differentiated tumors may show sheets of highly pleomorphic cells, while some angiosarcomas may appear relatively well-differentiated, sometimes placing a hemangioma in the differential diagnosis [1-4,6,7]. The heterogeneous cytoarchitectural features and sometimes high variation in the degree of differentiation seen in these tumors can make them a diagnostic challenge [1]. Several different immunohistochemical markers are used to identify angiosarcomas, although none have ideal sensitivity and specificity, and many are often expressed in non-vascular tissues [1-4,6,8-12]. Immunohistochemically, CD31 is the gold standard for angiosarcomas, being relatively specific and sensitive for these malignancies [1-4,6,7]. Factor VIII-related is the most specific immunostain but is also the least sensitive [1]. Other immunostains that have been employed in angiosarcoma are listed in Table 1.

**Table 1:** A list of the immunostains that have been used in the diagnosis of angiosarcomas. Co-expression of Factor VIII and CD31 is unusual in normal vessels [1-4,6,8-12]. IHC: Immunohistochemistry.

IHC marker	Comments
CD31	~80-90% positive in vascular tumors, high sensitivity and specificity, useful in poorly differentiated cases
CD34	~63% positive in vascular tumors
Factor VIII	~83% positive in vascular tumors, most specific, but least sensitive stain
ERG	~96% nuclear positivity in vascular tumors, a highly specific marker for benign and malignant vascular tumors
Fli-1	~94% nuclear positivity in vascular tumors, with 94% sensitivity and 100% specificity, equal, or exceeding that of CD31, CD34, and Factor VIII
VEGF	~94% positive in vascular tumors
Cytokeratin	~16-30% focally positive in epithelioid vascular tumors
EMA	~6% focally positive in epithelioid vascular tumors
CD30	~34% focally positive in epithelioid vascular tumors
D2-40/podoplanin	~43% positive in vascular tumors, suggests focal lymphatic differentiation
Ulex europaeus agglutinin 1	~70% positive in vascular tumors, useful in poorly differentiated cases

Approximately 30% of angiosarcomas show focal cytokeratin immunoreactivity and are designated as “epithelioid angiosarcomas,” as in the case presented here. Cytokeratin immunoreactivity should not lead to a misdiagnosis of a poorly differentiated carcinoma [1-4,6,8-12]. Another important differential diagnosis of epithelioid angiosarcoma is epithelioid sarcoma, especially pseudoangiosarcomatous growth pattern described in proximal-type epithelioid sarcoma [13]. At the molecular level, angiosarcomas show increased expression of the vascular-specific receptor tyrosine kinases, FLT1, TIE1, TEK, SNRK, and KDR, overexpression of the VEGF regulators HIF-1α and HIF-2α, amplified MYC in secondary angiosarcomas, recurrent angiogenesis-regulators PTPRB and PLCG1 mutations, FLT4 gene amplification in tumor with lymphatic differentiation, and, less commonly, CIC gene rearrangements in primary cutaneous angiosarcomas [1]. At the ultrastructural level angiosarcomas show cylindrical, single-membraned cytoplasmic, rod-shaped Weibel-Palade bodies, which store and release von Willebrand factor and P-selectin [1-4]. Presently there is no standardized treatment for primary pulmonary angiosarcomas. The present treatment modalities used in treatment include surgical resection, radio-, chemo-, and immune therapies, and various combinations of these therapies [4].

Due to their rarity and non-specific symptoms, primary pulmonary angiosarcomas are seldom in the initial differential diagnosis, with events such as thromboembolism, lung cancer, metastases, or pneumonia associated with infectious or autoimmunological etiologies [1,4]. Roughly 20% are asymptomatic and are identified incidentally at autopsy [4]. Most, as in the case presented here, do not have a history of tobacco use [2-4]. Additionally, most cases do not have histories of significant risk factors, although some cases have had prior histories of radiotherapy, radon, thorium dioxide, poly‑vinyl chloride, thorotrast, or copper mining dust exposures, or histories of a mastectomy or chronic empyema and tuberculosis [1-4]. The final diagnosis of the rare primary pulmonary angiosarcoma requires a careful analysis of the patient’s symptoms, combined with imaging investigation, and confirmation with histologic and immunohistochemical analyses.

## Conclusions

We reported a rare case of primary pulmonary epithelioid angiosarcoma in a middle-aged female patient. Primary pulmonary epithelioid angiosarcoma is an exceedingly rare and aggressive malignancy with a poor prognosis. Due to its rarity, diagnosis is often challenging and delayed, as it can mimic other more common pulmonary conditions both clinically and radiologically. Treatment options remain limited, with surgery, chemotherapy, and radiation therapy offering varying degrees of success, often dependent on the stage at which the disease is detected. Early and accurate diagnosis is crucial for improving outcomes, though the overall prognosis remains guarded. Continued research is essential to better understand the pathogenesis, optimize diagnostic approaches, and develop more effective therapies for this rare and devastating disease.

## References

[1] Cao J, Wang J, He C, Fang M (2019). Angiosarcoma: a review of diagnosis and current treatment. Am J Cancer Res.

[2] Krishnamurthy A, Nayak D, Ramshankar V, Majhi U (2015). Fluorine-18 fluorodeoxyglucose positron emission tomography/computed tomography in the detection of primary pulmonary angiosarcomas. Indian J Nucl Med.

[3] Khalid K, Khan A, Lomiguen CM, Chin J (2021). Clinical detection of primary pulmonary angiosarcoma. Cureus.

[4] Virarkar M, Tayyab S, Thampy R, Bhosale P, Viswanathan C (2019). Primary pulmonary angiosarcoma: case reports and review of the literature. Asian Cardiovasc Thorac Ann.

[5] Mandelstamm M (1923). About primary neoplasms of the heart (Article in German). Virchows Arch Path Anat.

[6] Ren Y, Zhu M, Liu Y, Diao X, Zhang Y (2016). Primary pulmonary angiosarcoma: three case reports and literature review. Thorac Cancer.

[7] Hsing JM, Thakkar SG, Borden EC, Budd GT (2007). Intimal pulmonary artery sarcoma presenting as dyspnea: case report. Int Semin Surg Oncol.

[8] Rao P, Lahat G, Arnold C (2013). Angiosarcoma: a tissue microarray study with diagnostic implications. Am J Dermatopathol.

[9] Miettinen M, Wang ZF, Paetau A, Tan SH, Dobi A, Srivastava S, Sesterhenn I (2011). ERG transcription factor as an immunohistochemical marker for vascular endothelial tumors and prostatic carcinoma. Am J Surg Pathol.

[10] Folpe AL, Chand EM, Goldblum JR, Weiss SW (2001). Expression of Fli-1, a nuclear transcription factor, distinguishes vascular neoplasms from potential mimics. Am J Surg Pathol.

[11] Ohsawa M, Naka N, Tomita Y, Kawamori D, Kanno H, Aozasa K (1995). Use of immunohistochemical procedures in diagnosing angiosarcoma. Evaluation of 98 cases. Cancer.

[12] Alimchandani M, Wang ZF, Miettinen M (2014). CD30 expression in malignant vascular tumors and its diagnostic and clinical implications: a study of 146 cases. Appl Immunohistochem Mol Morphol.

[13] Dey B, Srinivas BH, Badhe B, Nachiappa Ganesh R, Gochhait D, Toi PC, Jinkala S (2020). Malignant epithelioid soft tissue tumours- a pathologist's perspective with review of literature. Cureus.

